# Flanged males have higher reproductive success in a completely wild orangutan population

**DOI:** 10.1371/journal.pone.0296688

**Published:** 2024-02-09

**Authors:** Amy M. Scott, Graham L. Banes, Wuryantari Setiadi, Jessica R. Saragih, Tri Wahyu Susanto, Tatang Mitra Setia, Cheryl D. Knott

**Affiliations:** 1 Department of Anthropology, Boston University, Boston, Massachusetts, United States of America; 2 Department of Natural Resources and the Environment, University of New Hampshire, Durham, New Hampshire, United States of America; 3 Wisconsin National Primate Research Center, University of Wisconsin–Madison, Madison, Wisconsin, United States of America; 4 The Orang-Utan Conservation Genetics Project, Madison, Wisconsin, United States of America; 5 Eijkman Research Center for Molecular Biology, National Agency for Research and Innovation (BRIN), The Science and Technology Center of Soekarno, Cibinong, West Java, Indonesia; 6 Departemen of Biology, Faculty of Biology and Agricultural, Universitas Nasional, Kota Jakarta Selatan, Daerah Khusus Ibukota Jakarta, Indonesia; 7 Department of Biology, Boston University, Boston, Massachusetts, United States of America; Salim Ali Centre for Ornithology and Natural History, INDIA

## Abstract

Male orangutans (*Pongo spp*.) exhibit bimaturism, an alternative reproductive tactic, with flanged and unflanged males displaying two distinct morphological and behavioral phenotypes. Flanged males are larger than unflanged males and display secondary sexual characteristics which unflanged males lack. The evolutionary explanation for alternative reproductive tactics in orangutans remains unclear because orangutan paternity studies to date have been from sites with ex-captive orangutans, provisioning via feeding stations and veterinary care, or that lack data on the identity of mothers. Here we demonstrate, using the first long-term paternity data from a site free of these limitations, that alternative reproductive tactics in orangutans are condition-dependent, not frequency-dependent. We found higher reproductive success by flanged males than by unflanged males, a pattern consistent with other Bornean orangutan (*Pongo pygmaeus*) paternity studies. Previous paternity studies disagree on the degree of male reproductive skew, but we found low reproductive skew among flanged males. We compare our findings and previous paternity studies from both Bornean and Sumatran orangutans (*Pongo abelii*) to understand why these differences exist, examining the possible roles of species differences, ecology, and human intervention. Additionally, we use long-term behavioral data to demonstrate that while flanged males can displace unflanged males in association with females, flanged males are unable to keep other males from associating with a female, and thus they are unable to completely mate guard females. Our results demonstrate that alternative reproductive tactics in Bornean orangutans are condition-dependent, supporting the understanding that the flanged male morph is indicative of good condition. Despite intense male-male competition and direct sexual coercion by males, female mate choice is effective in determining reproductive outcomes in this population of wild orangutans.

## Introduction

Alternative reproductive tactics (ARTs) are the existence of two distinct phenotypes within one sex in the context of reproduction [1]. ARTs occur throughout the animal kingdom and are expected to evolve when there is strong sexual selection [1,2], specifically intra-sexual competition [2]. There are two primary explanations for the existence of ARTs. The two phenotypes may be either frequency-dependent evolutionary stable strategies, where the relative fitness of each morph depends on its frequency in the population [3] or condition-dependent, due to difference in the quality (i.e. age, body condition, experience, nutritional state and/or genes) of individuals where one morph is ‘making the best of a bad lot’ [4].

**Fig 1:**
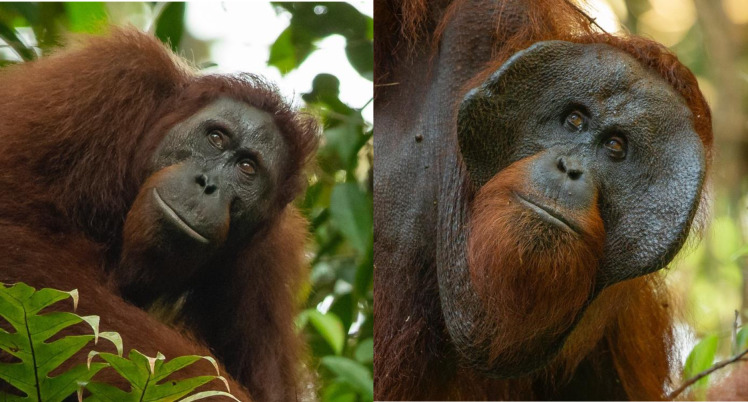
Example of male orangutans displaying the two alternative reproductive tactics. An unflanged male (left) lacks cheek pads and a throat sac and has a smaller body size. A flanged male (right) has secondary sexual characteristics including large cheek pads (flanges), a large throat sac, and larger body size. Photos by Tim Laman.

Male orangutans (*Pongo spp*.) display two ARTs with males exhibiting distinct morphological (Fig 1) (expressed as bimaturism) and behavioral phenotypes [5–8]. Flanged males (50–90 kg) are up to twice the size of unflanged males, but some unflanged males reach flanged male body size (30–59 kg) [9,10]. Flanged males possess secondary sexual characteristics, including an enlarged throat sac and cheek flanges [6,7,11], and are the only morph capable of producing long calls [5,6]. Flanged males are intolerant of each other, either avoiding or fighting and wounding each other [5,12], but they are typically more tolerant of unflanged males [5]. Conversely, unflanged males are generally tolerant of each other and tend to avoid flanged males [5,6]. Flanged males are dominant to unflanged males and displace unflanged males in consortships with females [5,6,12–14]. It has been suggested that flanged males use consortships to mate guard females, as a means to keep other males from mating with a female [12,15,16]. The male morphs also differ in activity patterns, with unflanged males traveling further per day than flanged males [14,17,18]. Due to these differences, the flanged male mating strategy has been described as “sit, call, and wait” and the unflanged male strategy described as “go, search, and find” [7,14].

For male orangutans, ARTs are plastic and sequential—an immature male first develops the unflanged male phenotype and may develop the flanged male phenotype later, but this transition is irreversible [7,19]. There is tremendous variation in the age of flange development, with wild males reportedly developing flanges from ages 14 to 30, and some males never developing flanges [11,16,20]. Flanged males in poor condition exhibit shriveled flanges and are referred to as past-prime males [21]. Past-prime males are not regularly seen, suggesting that this phase is not reached by all males, and is likely short for the males who do become past-prime. Additionally, the presence of past-prime males indicates that the flanged morph is so costly to maintain that some flanged males that cannot continue to maintain it enter the past-prime state [11].

Understanding how sexual selection acts on traits, such as ARTs, requires considering multiple mechanisms of sexual selection simultaneously [22]. Both male and female reproductive strategies are expected to impact the relative reproductive success of each morph [22], and this is especially true for primates, where male and female strategies are closely tied [23]. Orangutans are semi-solitary with large home ranges and adults primarily range alone or adult females range with dependent offspring [11,16,24,25], so reproduction first requires finding a mate. It has been suggested that one function of flanged male long calls is to attract females [26,27], and it may also play a role in male-male competition [26,28,29]. Across study sites, female orangutans prefer flanged males [5,6,21,30,31]. Orangutans also have slow life histories, including the longest interbirth interval of any mammal (7.6 years) [32,33]. Slow life histories push the potential for sexual conflict to an extreme [34]. Both male morphs employ sexual coercion in the form of forced copulations [30,35]. Sexual coercion can override female mate choice, but it is unknown if it increases male reproductive success. Female orangutans do not display overt signals of ovulation, such as the sexual swellings typical of many cercopithecoids [21,36] and ovulatory status appears to be effectively hidden from males [36,37]. Females preferentially mate with prime flanged males when they are ovulating and show increased willingness to mate with unflanged and non-prime males when the risk of conception is low [21]. Across primates this mating pattern—mating preferentially with preferred males when the likelihood of conception is highest and mating with non-preferred males when the likelihood of conception is lowest—is argued to be a paternity confusion strategy that reduces the likelihood of infanticide [21,38,39].

Quantifying the reproductive success of each morph is essential for testing hypotheses about the evolutionary pressures that resulted in orangutan ARTs. Previous studies of paternity in both Bornean (*Pongo pygmaeus*) and Sumatran (*Pongo abelii*) orangutans are limited by incomplete maternity data [40–43], the inclusion of ex-captive orangutans who may not display natural mating behaviors, or by provisioning from feeding stations and from veterinary care [13,20,44,45] (Table 1). The first orangutan (*P*. *abelli*) paternity study found that the two morphs had similar reproductive success and therefore concluded that the two morphs represent alternative mating strategies that coexist as evolutionary stable strategies [20]. The subsequent three orangutan (*P*. *pygmaeus*) paternity studies all concurred that flanged males had much higher reproductive success than unflanged males [13,44,45]. Each of these studies has unique limitations (Table 1). There are also important island or species differences to consider. *P*. *abelii* live in habitats with higher food availability, exist at higher densities, and are more social compared to *P*. *pygmaeus* [24,46]. We present paternity data from Cabang Panti Research Station in Gunung Palung National Park, Borneo, Indonesia (GPNP), the first from completely wild orangutans with known mothers. We compare our results against others to discern how study limitations and habitat differences explain contrasting results across sites.

**Table 1:** Paternity determination and paternity skew across study sites.

Study Site	No. offspring sired by flanged males	No. offspring sired by unflanged males	Total no. offspring with father assigned^a^	Total no. offspring tested	Total no. candidate sires tested^a^	Study Period^b^	Most successful male’s share (mean)	Most successful male’s share (range)	Limitation
Ketambe Research Station, Gunung Leuser National Park [18]	4	6	10	11	11	1983–1997 (11)	48.18	33.33–100	Ex-captives in study population
Kinabatangan Orang-utan Conservation Project, Lower Kinabatangan Wildlife Sanctuary [37]	9	1	10	16	16	1985–2000 (8)	33.32	18.18–50	Limited population knowledge; Mothers genetically assigned
Camp Leakey, Tanjung Puting National Park [36]	10	3^c^	14	25	17	1993–2009 (13)	56.57	14.29–100	Feeding station; Ex-captives in study population; veterinary care
Sepilok Orangutan Rehabilitation Center [12]	4	1	6^c^	8	4	2010–2014 (1)	57.14	NA	Feeding station; Ex-captives in study population; Only one flanged male sampled
Cabang Panti Research Station, Gunung Palung National Park	5	0	6^c^	13	20	2008–2014 (3)	33.33	20–40	
Total	32	11	46						

Gray background = *P*. *abelii*.

White background = *P*. *pygmaeus*.

^a^ = Number of offspring and candidate sires tested are likely an underestimate of the total number of offspring born or candidate sires in the study site due to sampling difficulties.

^b^ = Number in parentheses is the number of 5-year periods during the study period.

^c =^ Number of offspring sired by flanged males and unflanged males do not add up to the total because there was a male of unknown morph who sired an offspring.

Orangutan paternity studies also differ in the degree of male reproductive skew—the degree to which reproduction is monopolized versus shared (Table 1). Characterizing male reproductive skew is important for understanding the evolution of ARTs in orangutans. Across primates, the degree of male reproductive skew in multi-male groups is best explained by the degree of female reproductive synchrony and the number of males in the group [47,48]. The orangutan social system, with a high fission-fusion dynamic (social associations vary in size, composition, and cohesion) [11,24,49], and a lack of group formation, makes defining the number of males in a "group" difficult. However, there is clearly a male biased operational sex ratio, with many males competing for a few conception opportunities, due in part to the long interbirth interval [16]. In terms of female reproductive synchrony, reproduction is asynchronous, although some sites do see increases in births following periods of high fruit availability [50]. Even without female reproductive synchrony, in a dispersed social system, low male reproductive skew is expected [47]. Additionally, male dominance can lead to higher reproductive success through priority-of-access [51], but this is not the case for all species [52]. Here we compare male reproductive skew across sites and use long-term behavioral data to test the ability of flanged males or a single dominant flanged male to mate guard females.

We combine long-term behavioral observations and genetic paternity determination from a completely wild orangutan population at Cabang Panti Research Station in Gunung Palung National Park, Borneo, Indonesia, to investigate the evolution of male ARTs in Bornean orangutans. If male ARTs are frequency-dependent evolutionary stable strategies, we would expect the frequency of each morph to be stable and relative fitness of each morph to depend on its frequency in the population [1,3], i.e. if 20% of males are flanged then 20% of offspring will be sired by flanged males. Conversely if the male morphs are condition-dependent strategies, then we would expect unequal fitness benefits for each morph, where the morph in ‘poor condition’ has lower reproductive success and takes advantage of alternative tactics [1,4]. First, we determine the relative reproductive success of the two morphs and measure male reproductive skew. Second, we test the ability of flanged males to mate guard females. We then compare our results to those from prior studies in other populations to discern how study limitations and habitat differences might explain contrasting results across studies. Finally, we discuss the implications of these results for our understanding of the evolution of ARTs in orangutans and the interaction between male and female reproductive strategies.

## Materials and methods

### Study site and population

Orangutans (*Pongo pygmaeus wurmbii*) were studied in Gunung Palung National Park (GPNP), West Kalimantan, Indonesia, based out of the Cabang Panti Research Station (CPRS) (1°13´S, 1107´E) (3400 ha), as part of a study that began in 1994 [53]. Most orangutans encountered and followed were habituated and individually identifiable, but unknown and unhabituated individuals were also encountered, due to male dispersal and large home ranges [11,24,54,55]. Each month, phenology data were collected to characterize food availability of orangutan foods from 60 plots (totaling 9 ha) spread across 6 habitat types in the study site [56,57]. Fruit availability was calculated from the top 25 genera of plants that orangutans are known to consume most often at GPNP which represented 80% of fruit in their diet [57,58]. We then normalized that data by calculating modified Z scores from the percentage of stems that had mature or ripe fruits. Food availability was used as a control variable in our statistical models.

### Behavioral data collection

We used long-term data (2008–2019) from orangutans in CPRS collected during focal follows [59] to assess the ability of the two male morphs to effectively mate guard females and to create a male dominance hierarchy. During orangutan follows, an association was recorded whenever another orangutan came within 50 meters of the focal [60,61]. The identity and age-sex class of all orangutans was recorded. Males were classified by morph—flanged or unflanged. For this analysis, males who had small, developing flanges were classified as unflanged males. We used long-term follow data to tally the number of flanged and unflanged males that were seen in the study site one year prior to and following conception for each offspring, where we were able to identify a father and determine his morph. Females were classified as ‘sexually active’ or ‘non-sexually active’ based on the likelihood that they were fecund and actively mating. The ‘sexually active’ category included nulliparous females, parous females without dependent offspring, mothers with offspring over age six, and pregnant females in the first trimester. Females in this population are most proceptive to mating during the first trimester of pregnancy [21]. The non-sexually active category included parous females with dependent offspring under age six and pregnant females in the second and third trimester. Since orangutans have a gestation period of approximately eight months [62] and an average interbirth interval of 7.6 years [32], females will on average conceive when a dependent offspring is 6.8 years old and will begin mating 6–12 months before she conceives. Therefore, we used six years as a cut-off because we expected females to begin mating again at approximately that time. Additionally, we have previously shown that male-female interactions change when the dependent offspring reaches age six [63].

We analyzed all adult male-female associations from 2008–2019 (N = 759), noting the occurrence and outcome of an encounter with a second or ‘extra-pair male’ (EPM). If the association between the first male and female was terminated after the second male arrived, and the second male stayed with the female, we defined this as male displacement. Displacement did not necessarily involve agonism or aggression between the males, nor was it necessarily immediate. For each male-female association, the length of the association (in minutes) and all mating events were also recorded.

We analyzed all adult male-male interactions from 2008–2014, the period with both behavioral data and with paternity determination data, to evaluate male dominance rank. Offspring with known paternities were conceived from January 2010 to August 2014. During this period, nine of the sampled flanged males (Bilbo was still unflanged), an additional three individually recognizable flanged males, and up to seven unknown flanged males were observed in the study site. We examined the outcome of all dyadic interactions between flanged males to evaluate dominance rank. Dominance was defined by the outcomes of dyadic agonistic interactions [64]. We included avoidance, displacement, and chase interactions as dominance interactions with a clear dominant and subordinate individual.

### Sample collection

Fecal samples were collected after observed defecation from known and unknown orangutans from 2008 through 2019. When possible, two samples were collected from one individual on separate occasions. Samples from mother and dependent offspring were collected in the same encounter. Samples were stored in either RNAlater, 70% ethanol, or dried using the two-step ethanol alcohol-silica desiccation method [65,66]. Dried samples were stored at ambient temperature (up to 40° C) until analysis. Samples stored in RNAlater or 70% ethanol were stored at -20° C or -80° C.

### Genotyping and paternity analysis

We collected fecal samples from 42 orangutans for genotyping: 13 offspring, their 10 mothers, and 19 candidate fathers (8 unflanged, 10 flanged, and 1 observed as both unflanged and flanged males) in GPNP. Genomic DNA was extracted 2–3 times from each fecal sample using ChimerX stool DNA purification kits. Following Morin *et al*. [67], we quantified DNA content through qPCR^12^. We amplified a panel of 12 autosomal tetranucleotide microsatellites [20,44,68–71] (S1 Table). These were first co-amplified in an initial PCR reaction, with sufficient replicates to maintain error rates of less than 1% when scoring homozygotes, per Arandjelovic *et al*. [72], before the products were re-amplified with labelled primers in panels of 3–5 loci.

Fragment analysis was performed by the DNA sequencing unit at Eijkman Institute for Molecular Biology, using an Applied Biosystems 3130 Genetic Analyzer to size alleles against a GeneScan™ 500 LIZ™ internal size standard. Peaks were manually scored by two different people using GeneMapper (v3.7 and v4.0). Scores were concordant irrespective of software version. Heterozygotes were called when the same two alleles were observed in at least two independent amplifications, and homozygotes were called when only one allele was observed in up to five independent amplifications, per Arandjelovic *et al*. [72].

Prior to downstream analysis, CERVUS 3.0 [42] and MICRO-CHECKER 2.2.3 [73] were used to assess genotypes for null alleles, allelic dropout, and scoring errors due to stuttering, and to confirm that all 12 microsatellites were in Hardy-Weinberg equilibrium (S2 Table). Individual identity analysis was performed in CERVUS 3.0 to ensure that purported replicates derived from the same individual. Individuals genotyped at a minimum of nine loci were subsequently used in parentage analysis, having met the minimum number of loci needed to tell full siblings apart (PID-sibs <0.001) for the mean observed heterozygosity in our panel of microsatellites (*sensu* Waits *et al*. [74]).

Paternity analyses were performed in CERVUS 3.0 [42] and in COLONY 2.0.6.7 [75], using both an exclusionary approach and a likelihood approach. In the exclusionary approach, offspring are required to share one allele at each locus with the known mother and the other allele must be shared with the father. On the other hand, the likelihood approach in CERVUS 3.0 allows for genotyping errors, null alleles, and potential mutations. The advantage of COLONY 2.0.6.7 is that it uses a full-pedigree likelihood approach, rather than dyadic relationships, when inferring both parentage and sibship.

Field observation of mother-offspring pairs was confirmed using exclusionary maternity analysis CERVUS 3.0. Mothers were then used as known parents in CERVUS 3.0, increasing the statistical power of paternity assignment. All sampled males were considered candidate fathers for each offspring. Paternity was simulated using 100,000 offspring to obtain critical values of Delta at confidence levels of 80% (relaxed) and 95% (strict), *sensu* Marshall *et al*. [43]. For simulation in CERVUS 3.0, the proportion of candidate fathers sampled was inferred at three different values: 0.2, 0.5, 0.65 to simulate the possibility that an unsampled sire fathered offspring. The values 0.65 and 0.2 represent the upper and lower limits of ‘unknown’ males being entirely ‘known’ males or entirely ‘unknown, unique’ males, respectively. Each value produced the same results, so we report values using 0.5 as the proportion of candidate fathers.

In COLONY 2.0.6.7, analysis was run with the following parameters: female polygamy and male polygamy without inbreeding or clones, ‘long’ length of run, ‘high’ likelihood precision, no updating of allele frequency, and no sibship prior. Reported paternity results take known maternal genotype into account. Again, all sampled males were considered candidate fathers for each offspring.

### Cross-site comparisons

We compared our paternity data from CBRS in GPNP to published paternity results from four other orangutan study sites: Kinabatangan Orangutan Conservation Project, Lower Kinabatangan Wildlife Sanctuary [45]; Ketambe Research Station, Gunung Leuser National Park [20]; Camp Leakey, Tanjung Puting National Park [44]; and Sepilok Orangutan Rehabilitation Center [13].

### Reproductive skew

We calculated male reproductive skew (2008–2014) using two measures: Nonacs B index [76,77] and the most successful sire’s share as a percentage. We calculated both measures for our study population and the most successful sire’s share for all published orangutan paternity data. Due to the male dispersal and long lives, we were unable to accurately estimate adult male ages required for the multinomial skew index [78], and Nonacs B index was calculated with the Skew calculator 2013 (https://www.dropbox.com/home/2013%20Version, accessed December 2021) [79]. The B index takes residency and number of offspring into account (see S1 File for details of interpretation). We included only sampled males and the two unsampled fathers (of the two offspring for whom we could not identify a father) in our calculation. For the unsampled fathers, we used the average male residency time across this study period, 3 years.

### Statistical analysis

To test the ability of flanged and unflanged males to mate guard females, we used two-sided Fisher’s exact tests to compare the rate at which the two male morphs are displaced by an EPM in association with a female (N = 63). We additionally tested this hypothesis using two-sided Fisher’s exact tests to compare the rate at which the two male morphs are displaced by an EPM in association with only sexually active females (N = 36). Fisher’s exact tests are appropriate for comparisons when values in some categories are less than five [80].

Further, we tested the possibility that flanged male presence alone acts as a deterrent preventing EPM from encountering the flanged male-female pair using Chi-square tests of equal proportions and a binomial generalized linear mixed model (GLMM). We used Chi-square tests of equal proportions to compare the rate at which sexually active female (N = 486 associations), non-sexually active female (N = 201 associations), and total female associations (N = 706 associations) with flanged versus unflanged males encounter an additional or EPM. We also tested whether male-female association grouping (sexually active female-flanged male, sexually active female-unflanged male, non-sexually active female-flanged male, and non-sexually active female-unflanged male) impacted the chance of encountering an EPM using a binomial GLMM. Data exploration and model residuals revealed no violations of the assumptions of the binomial GLMM [81]. The response variable was the occurrence of an encounter with an EPM (yes/no). We used the length of a male-female association as an offset variable and included the identity of the male and female as random effects. Fruit availability (see Study Site and Population) was included as a fixed effect (control variable) because some study sites show that orangutans are more social during periods of high fruit [82–84] (but see [85,86]). We compared AIC values between models that excluded fixed effects to determine the best model and how to code male morph and female reproductive class (S3 Table).

We performed all statistical procedures in R [87]. For the nonparametric post-hoc tests, we used the package *PMCMR* [88]. For the binomial GLMMs, we used the packages *lme4* [89] and *arm* [90] to calculate confidence intervals. Graphs were made in the packages *ggplot2* [91] and *cowplot* [92].

This study followed the American Society of Primatologists’ ‘Ethical Treatment of Non-Human Primates’ principles. It was non-invasive and observational. All protocols were approved by The Eijkman Institute Research Ethics Commission, Boston University IACUC (protocol no. 11–045 and 14–043) or deemed exempt by Boston University IACUC. All protocols were approved by the Indonesian State Ministry for Research and Technology (RISTEK), the Ministry of Home Affairs and the Indonesian Institute of Sciences (LIPI), the Center for Research and Development in Biology (PPPB), and Balai Taman Nasional Gunung Palung (BTNGP). Sample collection was approved by Balai Taman Nasional Gunung Palung (BTNGP), permit numbers: 86/YPPN/SK/XII/2009-2019.

## Results

### Male reproductive success

Each of the three methods of paternity determination (exclusionary and likelihood approaches in CERVUS and full-pedigree likelihood approach in COLONY) were concordant in paternity assignment (Table 2). Paternity could be assigned for five out of seven offspring conceived during the sampling period (2008–2019) and one individual conceived prior to the sampling period. The flange-status of this sire at the time of conception (ca. 2005) is unknown, but he was flanged at first observation in 2009. Over a six-year period (2009–2014), four flanged males sired five offspring, indicating that male morph plays an important role in male reproductive success (Table 2). During each of these conception periods, there were never more flanged males than unflanged males observed in the study site (S4 Table). The mothers of these five offspring were parous at the time of conception. For the two offspring for whom fathers could not be determined, the COLONY pedigree results inferred different fathers.

**Table 2:** Paternity assignment at cabang panti research station in GPNP.

			CERVUS					COLONY	
			Exclusion			Likelihood		Prob.	
Offspring	Est. Birth Year	Mother	Trio mis-match	P_e_	Next best mis-match	Delta			Assigned Father
Dagul	2002	Delly	⎻	⎻	⎻	⎻		⎻	⎻
Rossa	2004	Veli	⎻	⎻	⎻	⎻		⎻	⎻
Berani	2005	Bibi	0	0.999	4	13.7	‡	0.999	Codet
Ijal	2005	Irma^a^	⎻	⎻	⎻	⎻		⎻	⎻
Telur	2007	Tari	⎻	⎻	⎻	⎻		⎻	⎻
Uok	2007	Umi	⎻	⎻	⎻	⎻		⎻	⎻
Januari	2009	JT	⎻	⎻	⎻	⎻		⎻	⎻
Benny	2010	Beth	0	0.999	3	7.76	‡	0.961	**Prabu**
Dolia	2011	Dewi	0	0.999	2	4.30	‡	0.023	**Senja** ^a^
Hannah	2012	Hera^a^	⎻	⎻	⎻	⎻		⎻	⎻
Vanna	2012	Veli	0	0.999	1	6.50	‡	0.880	**Prabu**
Tawni	2014	Tari	1	0.999	4	4.08	‡	0.023	**Manda** ^**b**^
Bayas^a^	2015	Bibi	0	0.998	2	5.29	‡	0.072	**Moris**

Gray = conceptions within the sampling (fecal and behavioral data collected) period.

^a^ = genotyped at 11 loci, not all 12 loci.

^b^ = genotyped at 10 loci, not all 12 loci.

**Bold** = male is known to have been flanged at the time of conception. Non-bolded father means that his phenotype was unknown at the time of conception.

Trio mismatch = the number of loci that are a mismatch in the trio of offspring, mother and assigned father.

P_e_ = exclusion probability, calculated in CERVUS 3.0 using allele frequencies from all 48 individuals genotyped.

Next best mismatch = refers to the trio of offspring, mother, and the male with the closest match after the assigned father.

‡ = the trio delta value meets the strict (95%) confidence level.

### Male reproductive skew

We found low reproductive skew in our study population. From 2008 to 2014, the period with both behavioral data and offspring genetic sampling, the most successful sire’s share was 28.57% and Nonacs B index was 0.0004 (N_potential sires_ = 17, N_offspring_ = 6, P = 0.502, 95% CI = -0.121–0.188) (Table 1). Our Nonacs B values indicated either a random or equal distribution of male reproductive skew (equalB = 0.121, monopolyB = 0.922, see S1 File for details of interpretation). Unfortunately, we were not able to construct a male dominance hierarchy because only six interactions between flanged males were observed during this same period, with 3434 flanged male observation hours (S5 Table). Even with few observations, we did not find a strict relationship between male dominance and reproductive success. For example, Senja, who sired one known offspring during this period, was subordinate to Codet, who did not sire any known offspring during this period (Tables 1 and S5).

### Cross-site comparisons

Combining paternity assignment data across the five study sites showed that, overall, flanged males sired a greater proportion of offspring (69.57%) than did unflanged males (23.91%) (Table 1). This was especially true for Bornean sites, where flanged males sired 77.78% of offspring (Table 1). There was also variation in the degree of male reproductive skew across study sites (Table 1). Across study sites, the mean most successful sire’s share (for 5-year periods) ranged from 33.33%-57.57% (Table 1).

### Mate guarding

Our paternity results demonstrated that a single male was unable to monopolize paternity within our study site during any time period. To examine this from a behavioral perspective, we examined the ability of males to mate-guard females. We tested whether the presence of a male in association with a female served to deter a second or ‘extra-pair male’ (EPM) from interacting with that female. Further, we examined the outcome of those interactions to determine if flanged males were able to displace unflanged males.

**Fig 2:**
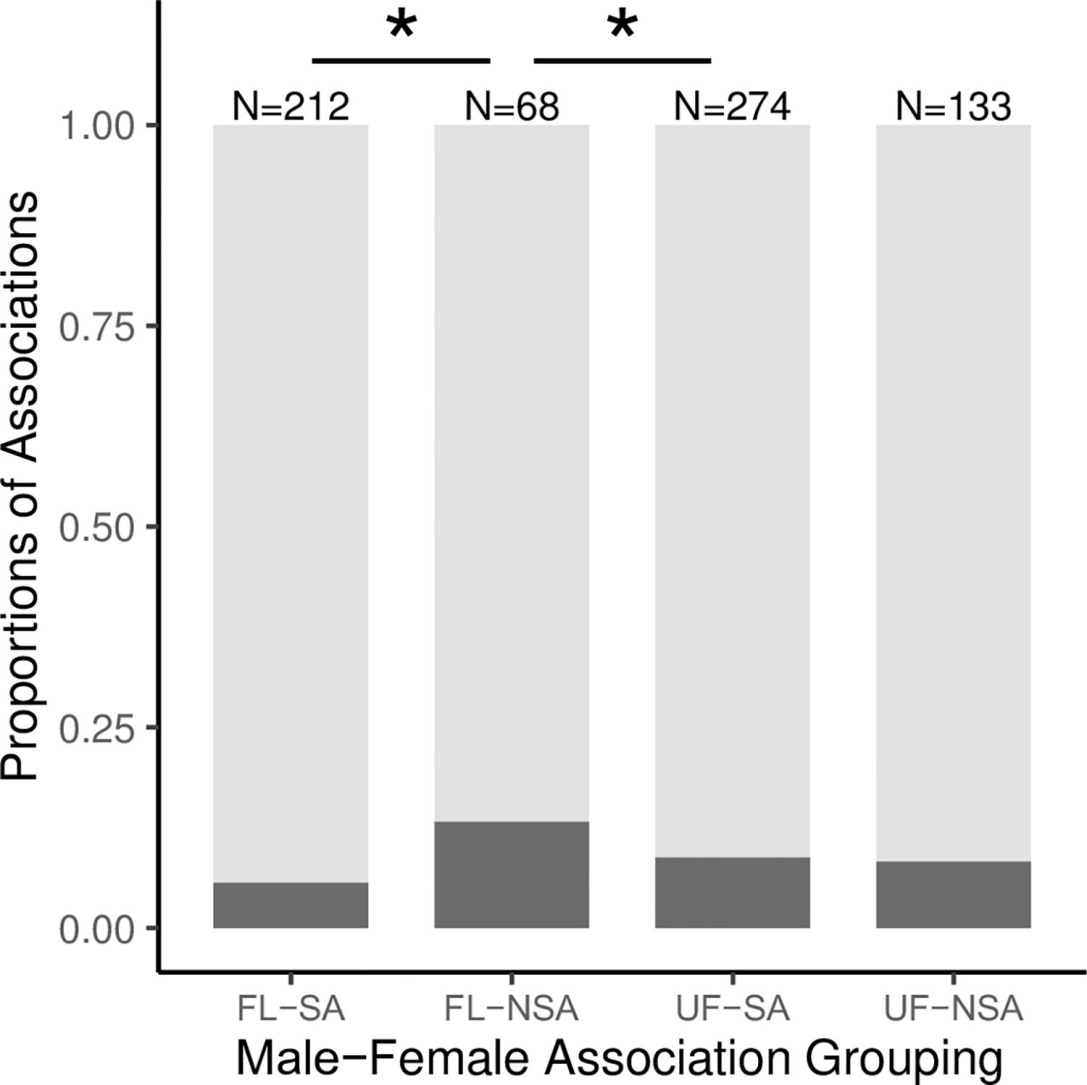
The proportion of male-female associations in which the dyad encounters an ‘Extra-Pair Male’ (EPM) by male-female association group type. FL-SA = flanged male/sexually active female association. FL-NSA = flanged male/non-sexually active female association. UF-SA = unflanged male/sexually active female association. UF-NSA = unflanged male/non-sexually active female association. N values at the top of each column show the number of male-female associations in each group type. Dark gray represents encounters with an EPM and light gray represents no encounter with an EPM. Significance values from the binomial GLMM (* = P < 0.05).

We observed no significant difference in the rate at which female associations with flanged and unflanged males encountered an EPM (χ^2^ = 0.120, df = 1, P = 0.730, N = 706). On average, an EPM was encountered every 56.65 hours of unflanged male-female associations, and every 54.08 hours of flanged male-female associations (S6 Table). Likewise, there was not a significant difference in the rate at which sexually active female associations (χ^2^ = 2.412, df = 1, P = 0.120, N = 486) or non-sexually active female associations (χ^2^ = 0.746, df = 1, P = 0.388, N = 201) with flanged and unflanged males encountered an EPM. On average, sexually active females in association with flanged males encountered an EPM every 95.85 hours and in association with unflanged males encountered an EPM every 70.78 hours (S6 Table). In contrast, non-sexually active females in association with flanged males encountered an EPM every 15.28 hours and in association with unflanged males encountered an EPM every 43.08 hours, on average (S6 Table). Our best binomial Generalized Linear Mixed Model (GLMM) found that food availability and both male and female age-sex classes significantly impacted the likelihood that a male-female association would encounter an EPM (S7 Table). Flanged males with non-sexually active females were significantly more likely to encounter an EPM than were either flanged or unflanged males with sexually active females (S7 Table and Fig 2).

**Fig 3:**
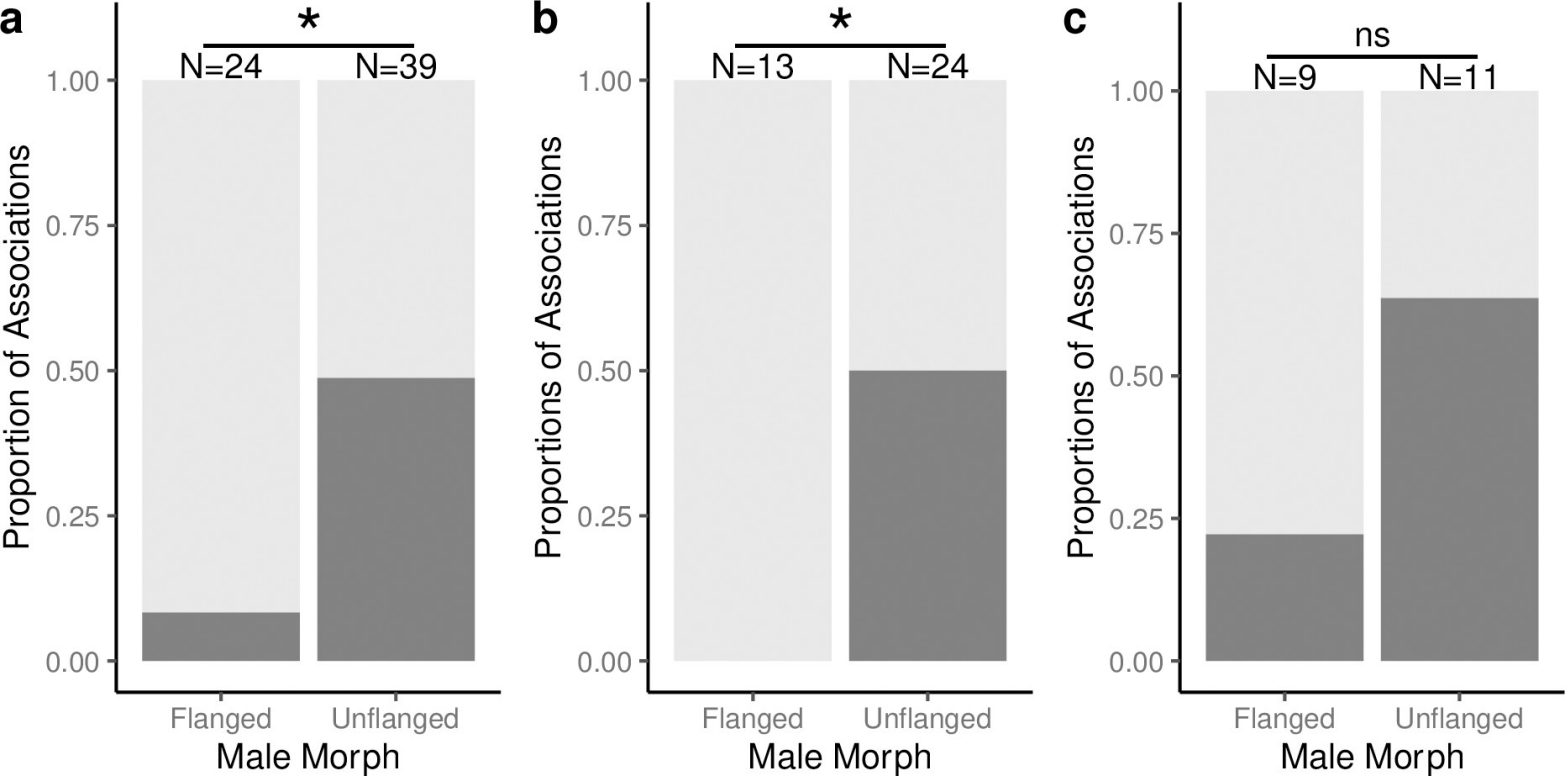
Male displacement in male-female associations. The proportion of (a) all male-female associations, (b) a subset of male-female associations where the female is a sexually active, and (c) a subset of male-female associations where the female is a non-sexually active in which the first male is displaced by an ‘extra-pair male’ (EPM). Male displacement is represented by darker shading. N values at the top of each column show the number of male-female associations by male morph. Dark gray represents encounters with displacement and light gray represents encounter with no displacement. Significance values from Fisher’s exact tests (* = P < 0.05, ns = P > 0.05).

However, after an EPM was encountered, there was a statistically significant difference between male morphs in the proportion of encounters in which the first male associating with a female was displaced (Fisher’s Exact Test, N = 50, P = 0.0004) (Fig 3A). Unflanged males were displaced in 60% of encounters with an EPM. Of the 18 times that unflanged males were displaced, 61% of the EPM were flanged. Flanged males were only displaced in 10% of encounters with an EPM and they were never displaced by unflanged males. This 10% represents one instance in which one flanged male chased off another flanged male in the presence of two non-sexually active females. When considering only sexually active females, flanged males were statistically significantly less likely to be displaced than unflanged males (Fisher’s Exact Test, N = 36, P = 0.003) (Fig 3B). Conversely, when considering only non-sexually active females, there is no difference in the rate of displacement between male morphs (Fisher’s Exact Test, N = 20, P = 0.379) (Fig 3C). Thus, both female reproductive state and male morph are important determinants of orangutan mating behavior [21].

## Discussion

### Male reproductive success and skew

Our paternity results (6 assigned paternities over 10 years) most closely align with those of Kinabatangan [45], finding low reproductive skew among flanged males. While Kinabatangan inferred maternity from genetic data, our results confirm the same overall pattern. Only these two studies are from wholly wild and unprovisioned orangutans in primary rainforest habitat without feeding stations, ex-captive orangutans, or veterinary care. This suggests that flanged males have higher reproductive success than unflanged males in completely wild Bornean populations, and that a single flanged male cannot monopolize paternity. In contrast, provisioning from feeding stations at Tanjung Putting [44] and Sepilok [13], in conjunction with veterinary interventions, may explain why a single flanged male was able to monopolize paternity at these two sites. Feeding stations may create an unnaturally high concentration of female orangutans in one area, increasing the ability of a single male to monopolize females. One unexpected outcome of feeding stations may be a reduction in genetic diversity in subsequent generations due to high male reproductive skew. It is likely that without feeding stations, either (1) dominant males are unable to monopolize females across large areas or (2) male dominance hierarchies are less strict when males are not competing over access to a feeding station. Due to the rarity of interactions between flanged males (6 interactions in 7 years), we could not construct a dominance hierarchy, but all observed interactions suggest a linear hierarchy with no observations of rank reversals or rank instability. Continued study of paternity and male interactions at GPNP and more orangutan study sites could differentiate between these two possibilities.

Paternity results from Ketambe, Sumatra [20], starkly contrast with those from Borneo—unflanged males at Ketambe had higher reproductive success than flanged males [20]. It is unclear if this represents a species difference or unusual population parameters. The combination of male rank instability, first-time mothers, and ex-captive females in that study [20,93] may have resulted in an inflated reproductive advantage for unflanged males. For instance, Sepilok and Tanjung Puting also found that the offspring of first-time mothers were sired by unflanged males [13,44], although in GPNP nulliparous females formed preferential mating relationships with flanged males [94]. If there truly is a species difference between the relative reproductive success of flanged and unflanged males, it is likely due to differences in the duration of the unflanged stage and variation in the relative proportions of each morph between the islands [19,86].

However, it is important to note that orangutan paternity studies are limited by small sample sizes (Table 1) due to their long interbirth intervals and semi-solitary social structure. Smaller samples are more subject to random stochasticity, which may also play a factor in explaining the differences between sites, but comparison of data across five different sites adds robustness to these comparisons. Small sample sizes may contribute to the finding of lower reproductive skew. In this comparative perspective, the two Bornean sites with completely wild orangutans (GPNP and Kinabatangan) agree that flanged males have higher reproductive success than unflanged males and reproductive success is spread broadly across many flanged males. But with only one Sumatran site in the sample [20], where there are also ex-captives, it is unclear if that pattern holds for Sumatran orangutans. Half of the offspring (5 out of 10) in Ketambe were born to matrilines with ex-captive mothers, and 4 of these 5 offspring were sired by unflanged males [93]. If mating strategies are learned through the observation of the mother, then ex-captive matrilines might not display the same mate choice preferences as wild orangutans.

### Orangutan reproductive strategies

Orangutans exhibit male-male competition, sexual coercion by males, and female mate choice. Reproductive success is impacted by the interaction between each of these male and female reproductive strategies. Due to the highly dispersed spatial distribution of female orangutans [11,24,25], it is expected that a single male cannot monopolize females or conceptions, resulting in low male reproductive skew [47]. Our paternity data concluded that a single male cannot monopolize paternity, and the behavioral data on male-female association encounter rates with EPMs further addresses the inability of a single flanged male to monopolize females. Consistent with other studies [6,16,30], we found that flanged males at GPNP were able to displace unflanged males associating with females, specifically with sexually active females. However, the mere presence of a flanged male with a female does not keep other males away. But once a sexually active female is an association with a male, regardless of male morph, the pair is less likely to encounter an EPM. Female orangutans at GPNP display a mixed mating strategy preferentially associating with prime, flanged males when they are most likely to conceive and with non-prime, unflanged males when they are less likely to conceive [21,38]. Thus, females may be choosing who to associate with based on their probability of conception [21,38]. The limited ability of flanged males to mate guard further highlights the importance of female choice in facultative associations and mating. Therefore, female preference for flanged males, coupled with the flanged male ability to displace unflanged males, operate in parallel leading to higher reproductive success for flanged males, and flanged male inability to completely mate guard females, leads to low reproductive skew among these flanged males. Because both flanged and unflanged males perform forced copulations [11,35], our results cannot speak to the efficacy of that form of sexual coercion in leading to reproductive success. Since unflanged males have lower reproductive success, it appears that harassment by unflanged males is not a successful reproductive strategy. Instead, female preference for flanged males and flanged male competitive ability are operating in the same direction, leading to higher reproductive success for flanged males.

### Alternative reproductive strategies

Our results also have important implications for understanding ARTs in male orangutans. ARTs have been hypothesized to be either frequency-dependent evolutionary stable strategies [3] or due to difference in the quality of individuals [1,4]. The first published study of orangutan paternity, and still the only study in Sumatran orangutans, found that the two morphs had comparable reproductive success at Ketambe, and thus argued that the two morphs were evolutionary stable strategies [7,20]. Now, 20 years later with data from an additional four Bornean sites, it is clear, that at least in Borneo, flanged males have higher reproductive success than unflanged males.

The relative numbers of flanged and unflanged males are pivotal to our interpretation of paternity data, but accurate counts of the numbers of males in a study site are difficult to obtain due to large home ranges and the difficulty of visually identifying orangutans who transition from unflanged to flanged males. Cross-site comparisons agree that in Sumatra there are approximately twice as many unflanged males as flanged males, whereas in Borneo there is more inter-site variation, but the morphs exist in approximately equal proportions [12,19]. Long-term demographic data at GPNP agrees with these approximations [35]. These proportions indicate that males remain in the unflanged morph longer in Sumatra than in Borneo [19]. Because the first paternity study found that unflanged males sired 60% of offspring in a Sumatran population, it was argued that flanged and unflanged male morphs represent alternative mating strategies that coexist, representing evolutionary stable strategies [7,20]. If this result is representative of all of Sumatra, it could explain why Sumatran males remain unflanged for longer than Bornean males. In this case, unflanged males avoid the energetic and competitive costs of becoming a flanged male [11], while achieving reproductive success. However, the paternity data from Borneo does not support the understanding that male alternative reproductive strategies are frequency-dependent evolutionary stable strategies. In Borneo, 77% of offspring are sired by flanged males while flanged males only represent approximately 50% of all males, demonstrating that the flanged morph is absolutely and relatively more successful. Because the reproductive success of the morphs is not related to their proportion in the population, Bornean orangutan ARTs are not frequency-dependent.

For Bornean orangutans, ARTs are due to individual differences in quality, with the flanged morph indicating higher quality and unflanged morph indicating lower quality. Our results support the view that the unflanged morph is a transitional stage, where unflanged males are in a ‘waiting room’, avoiding the costs associated with the flanged morph, and ‘making the best of a bad situation’ until they are able to flange [7,93]. The variation in results at different study sites highlights the dynamic nature of ARTs; the ability of each morph to attain reproductive success is likely highly dynamic, depending on the relative proportions of each male morph and density of orangutans, which is influenced by food availability [24,95]. More data on the relative proportion of each male morph, male dominance hierarchies, and paternity data from additional study sites of *P*. *abelii* in Sumatra will clarify if island differences are due to ecological factors or if there are true species differences. Additionally, studies of the recently described Tapanuli orangutan (*Pongo tapanuliensis*)—who live on Sumatra, south of Lake Toba, but inhabit less productive forests, live at lower densities, and are less social and thus more similar to Bornean orangutans than Sumatran orangutans [84]—will further help to clarify the relative roles of ecology and species differences.

These combined paternity results across sites align with the model of developmental arrest in male orangutans developed by Pradhan *et al*. [95] which explains differences in the ratio of flanged to unflanged males across sites through ecology. According to this model, longer delays in the development of flanges are expected when females are monopolizable by the dominant male because, in this situation, non-dominant flanged males will have lower reproductive success; thus, males should remain unflanged to avoid the costs of the flanged male morph [24,46,95]. Dominant male monopolization of females is expected when orangutans live at higher densities and are more gregarious, which is related to increased food availability [24,46]. Thus, where orangutans live at higher densities (i.e., Ketambe, Sumatra), a dominant flanged male is expected to be able to monopolize females, and a smaller proportion of flanged males are expected. It is worth noting that at Ketambe, there are periods of both high reproductive skew, where a single flanged male sires many offspring, and periods of low reproductive skew which correspond to times of rank instability. Conversely, where orangutan habitat is less productive and orangutans live at lower densities (i.e., Borneo), a dominant flanged male is not expected to be able to monopolize females, and a greater proportion of flanged males are expected. Paternity results from the two Bornean sites with completely wild orangutans agree with this model, showing that in the absence of feeding stations, a single male is not able to monopolize females. And in the Bornean case, we also see short developmental arrest, resulting in relatively more flanged males. In Borneo, with low reproductive skew among flanged males, there is a reproductive benefit to flanging.

Despite intense male-male competition [5,12,96] and sexual coercion [11,35], female choice remains an important factor in determining orangutan reproductive outcomes. The importance of female choice may explain why it is that in all primate species with male ARTs, the morphs are attributed to individual differences in quality [23]. In taxa where the costs of reproduction are disproportionately borne by one sex, we expect strong sexual selection, including mate choice to evolve [97]. In the case of mammals where obligate female gestation and lactation mean that females must invest heavily in reproduction and parental care, we expect female choice to evolve [98], and thus is not surprising that male ARTs in orangutans are signals of male quality that are subject to female choice. We predict that in species with both strong mate choice, driven by differential costs of reproduction, and ARTs, the ARTs will be condition-dependent, rather than frequency-dependent.

This study of orangutan paternity determination in GPNP is the first study of orangutan paternity from a completely wild population in a primary rainforest site, without feeding stations, rehabilitant orangutans, or veterinary care, and with known maternal-offspring relationships. This enables us to better understand why previous orangutan paternity studies disagree on which morph has higher reproductive success and the degree of male reproductive skew. We show that the ARTs are condition-dependent. Flanged males have higher reproductive success, and unflanged males are ‘making the best of a bad situation’.

## Supporting information

S1 Table Panel of microsatellite primers used for genotyping. (DOCX)

S2 Table Summary statistics for the 12 microsatellite loci used. (DOCX)

S3 Table GLMM comparisons. (DOCX)

S4 Table Ratio of flanged to unflanged males during each of the five conception periods where paternity and male morph were determined. (DOCX)

S5 Table Flanged male-flanged male dyadic dominance interactions from 2008 to 2014. (DOCX)

S6 Table Rates at which male-female associations encounter an additional or Extra-Pair Male (EPM), expressed as the average number of hours of association per encounter with an EPM. (DOCX)

S7 Table GLMM testing the probability that a male-female pair encountered an EPM. (DOCX)

S1 File Reproductive skew calculation and nonac’s b interpretation. (DOCX)
