# Supplementary material for: Flanged males have higher reproductive success in a completely wild orangutan population
Source: PLoS One. 2024 Feb 9;19(2):e0296688. doi: 10.1371/journal.pone.0296688 (PMC10857694; doi:10.1371/journal.pone.0296688)
Supplement: S1 Table — (DOCX) [file pone.0296688.s001.docx]

**S1 Table.** **Panel of microsatellite primers used for genotyping**

| Locus | Label | Forward Primer Sequence  (5’ to 3’) | Reverse Primer Sequence  (3’ to 5’) |
| --- | --- | --- | --- |
| D1S550 [70] | VIC | CCTGTTGCCACCTACAAAAG | TAAGTTAGTTCAAATTCATCAGTGC |
| D2S1326 [70] | 6FAM | AGACAGTCAAGAATAACTGCCC | CTGTGGCTCAAAAGCTGAAT |
| D3S2459 [70] | NED | CTGGTTTGGGTCTGTTATGG | AGGGACTTAGAAAGATAGCAGG |
| D4S1627 [70] | PET | AGCATTAGCATTTGTCCTGG | GACTAACCTGACTCCCCCTC |
| D4S2408 [70] | VIC | AATAAACTTCAACTTCAATTCATCC | AGGTAAAGGCTCTTCTTGGC |
| D5S1457 [68] | 6FAM | TAGGTTCTGGGCATGTCTGT | TGCTTGGCACACTTCAGG |
| D5S1470 [68] | 6FAM | CATGCACAGTGTGTTTACTGG | TAGGATTTTACTATATTCCCCAGG |
| D6S501 [70] | 6FAM | GCTGGAAACTGATAAGGGCT | GCCACCCTGGCTAAGTTACT |
| D12S375 [20] | VIC | TTGTTGAGGGTCTTTCTCCA | TCTTCTTATTTGGAAAAGTAACCC |
| D13S321 [70] | NED | TACCAACATGTTCATTGTAGATAGA | CATACACCTGTGGACCCATC |
| D13S765 [70] | VIC | TGTAACTTACTTCAAATGGCTCA | TTGAAACTTACAGACAGCTTGC |
| O4_B6 [68] | 6FAM | TGGAGCCTGAATATGTGACTGAAT | AATGCCAGGATTTCCTTCTTTTT |
| AMEL [71] | VIC | ACCACCAGCTTCCCAGTTTA | GCTGGGWTAGAACCAAGCTG |
| SRY [71] | 6FAM | AGTGAAGCGACCCATGAACG | TGTGCCTCCTGGAAGAATGG |

Forward and reverse sequences for primers used in the multiplex genotyping panel. All primers have been previously published. In the second multiplex reaction, forward primers were fluorescently labeled. Table modified from Banes et al. (2015) [44].
