# Supplementary material for: Flanged males have higher reproductive success in a completely wild orangutan population
Source: PLoS One. 2024 Feb 9;19(2):e0296688. doi: 10.1371/journal.pone.0296688 (PMC10857694; doi:10.1371/journal.pone.0296688)
Supplement: S2 Table — (DOCX) [file pone.0296688.s002.docx]

**S2 Table.** **Summary statistics for the 12 microsatellite loci used**

| Locus | No. of Alleles | No. of Individuals Genotyped | No. of Hetero-zygotes | No. of Homo-zygotes | H_obs_ | H_exp_ | HWE-p |  |
| --- | --- | --- | --- | --- | --- | --- | --- | --- |
| D1S550 | 4 | 48 | 38 | 10 | 0.792 | 0.742 | ⎼ | |
| D2S1326 | 4 | 46 | 26 | 20 | 0.565 | 0.575 | 0.6619 | |
| D3S2459 | 6 | 48 | 32 | 16 | 0.667 | 0.682 | 0.9410 | |
| D4S1627 | 3 | 48 | 30 | 18 | 0.625 | 0.620 | 0.8423 | |
| D4S2408 | 5 | 47 | 30 | 17 | 0.638 | 0.593 | 0.3915 | |
| D5S1457 | 7 | 48 | 39 | 9 | 0.813 | 0.700 | 0.0433 | |
| D5S1470 | 5 | 47 | 37 | 10 | 0.787 | 0.721 | 0.2073 | |
| D6S501 | 6 | 47 | 33 | 14 | 0.702 | 0.779 | 0.7066 | |
| D12S375 | 4 | 47 | 37 | 10 | 0.787 | 0.598 | 0.0028 | |
| D13S321 | 3 | 47 | 30 | 17 | 0.638 | 0.586 | 0.6372 | |
| D13S765 | 3 | 48 | 24 | 24 | 0.500 | 0.483 | ⎼ | |
| O4 B6 | 8 | 48 | 37 | 11 | 0.771 | 0.737 | 0.683 | |

H_obs_ is the observed heterozygosity value and H_exp_ is the expected heterozygosity value. HWE-p is the p values for deviation from Hardy-Weinberg equilibrium. P > 0.004 indicates the locus is in Hardy-Weinberg equilibrium and (–) means that Hardy-Weinberg equilibrium was unable to be calculated.
