# Supplementary material for: Flanged males have higher reproductive success in a completely wild orangutan population
Source: PLoS One. 2024 Feb 9;19(2):e0296688. doi: 10.1371/journal.pone.0296688 (PMC10857694; doi:10.1371/journal.pone.0296688)
Supplement: S3 Table — (DOCX) [file pone.0296688.s003.docx]

**S3 Table.** **GLMM comparisons**

| Model | Male Type Estimate | Female Type Estimate | Interaction Term Estimate | Fruit Availability Estimate | AIC | △AIC | Weighted AIC | |
| --- | --- | --- | --- | --- | --- | --- | --- | --- |
| 1 | NA | NA | *(combo)* | *0.4831* | 425.7 | ⎻⎻ | 0.29929 |  |
| 2 | 0.568 | *1.9726* | *-1.516* | *0.4831* | 425.7 | 0 | 0.29929 |  |
| 3 | ⎻⎻ | 1.0747 | ⎻⎻ | *0.5151* | 425.9 | 0.2 | 0.27081 |  |
| 4 | *-0.081* | 1.0761 | ⎻⎻ | *0.5134* | 427.9 | 2.2 | 0.09962 |  |
| 5 | ⎻⎻ | ⎻⎻ | ⎻⎻ | *0.4528* | 430.9 | 5.2 | 0.02223 |  |
| 6 | -0.048 | ⎻⎻ | ⎻⎻ | *0.45195* | 432.9 | 7.2 | 0.00818 |  |
| 7 | 0.514 | 1.8822 | -1.989 | ⎻⎻ | 440.0 | 14.3 | 0.00023 |  |
| 8 | NA | NA | *(combo)* | ⎻⎻ | 440.0 | 14.3 | 0.00023 |  |
| 9 | ⎻⎻ | 0.6561 | ⎻⎻ | ⎻⎻ | 443.3 | 17.6 | 0.00005 |  |
| 10 | ⎻⎻ | ⎻⎻ | ⎻⎻ | ⎻⎻ | 443.9 | 18.2 | 0.00003 |  |
| 11 | -0.357 | 0.6699 | ⎻⎻ | ⎻⎻ | 445.0 | 19.3 | 0.00002 |  |
| 12 | -0.308 | ⎻⎻ | ⎻⎻ | ⎻⎻ | 445.7 | 20.0 | 0.00001 |  |

Significant effects are shown in *italics*

| 1. Encounter ~ Male-Female type combo + Fruit + offset: length of association + random: maleID + random: female ID |
| --- |
| 2. Encounter ~ Male type * Female type + Fruit + offset: length of association + random: maleID + random: female ID |
| 3. Encounter ~ Female type + Fruit + offset: length of association + random: maleID + random: female ID |
| 4. Encounter ~ Male type + Female type + Fruit + offset: length of association + random: maleID + random: female ID |
| 5. Encounter ~ Fruit + offset: length of association + random: maleID + random: female ID |
| 6. Encounter ~ Male type + Fruit + offset: length of association + random: maleID + random: female ID |
| 7. Encounter ~ Male type * Female type + offset: length of association + random: maleID + random: female ID |
| 8. Encounter ~ Male-Female classes + offset: length of association + random: maleID + random: female ID |
| 9. Encounter ~ Female type + offset: length of association + random: maleID + random: female ID |
| 10. Encounter ~ offset: length of association + random: maleID + random: female ID |
| 11. Encounter ~ Male type + Female type + offset: length of association + random: maleID + random: female ID |
| 12. Encounter ~ Male type + offset: length of association + random: maleID + random: female ID |
