# Supplementary material for: Flanged males have higher reproductive success in a completely wild orangutan population
Source: PLoS One. 2024 Feb 9;19(2):e0296688. doi: 10.1371/journal.pone.0296688 (PMC10857694; doi:10.1371/journal.pone.0296688)
Supplement: S4 Table — (DOCX) [file pone.0296688.s004.docx]

**S4 Table. Ratio of flanged to unflanged males during each of the five conception periods where paternity and male morph were determined.**

| Offspring | Sire^a^ | Conception Period^b^ | Observed number of males^c^ | Observed ratio of flanged to unflanged males |
| --- | --- | --- | --- | --- |
| Benny | **Prabu** | 1/17/2009 – 1/17/2011 | 45 | 0.591 |
| Dolia | **Senja** | 5/1/2009 – 5/1/2011 | 30 | 0.579 |
| Vanna | **Prabu** | 5/1/2010 – 5/1/2012 | 13 | 0.625 |
| Tawni | **Manda** | 11/1/2012 – 11/1/2014 | 24 | 1.000 |
| Bayas | **Moris** | 7/18/2013 – 7/18/2015 | 18 | 0.636 |

^a^ Bold signifies a flanged male

^b^ The conception period is one year before and one year after the estimated date of offspring conception. We used a large window of time because it can be months between male sightings due to large home ranges.

^c^ This number represents a maximum, it assumes that unknown males observed on different occasions are unique individuals. This is likely an overestimate of the true number of unique males observed in the study site. Since both the number of flanged and unflanged males are over-estimates, the ratio should still be realistic.
