# Supplementary material for: Flanged males have higher reproductive success in a completely wild orangutan population
Source: PLoS One. 2024 Feb 9;19(2):e0296688. doi: 10.1371/journal.pone.0296688 (PMC10857694; doi:10.1371/journal.pone.0296688)
Supplement: S5 Table — (DOCX) [file pone.0296688.s005.docx]

**S5 Table. Flanged male-flanged male dyadic dominance interactions from 2008 to 2014**

| Dominant Male | Subordinate Male | Behavior | Female present | Date |
| --- | --- | --- | --- | --- |
| Codet | Senja | chase | yes | 12/24/2009 |
| Prabu | Unknown^a^ | chase | no | 12/29/2009 |
| Unknown^a^ | Codet | avoid | yes | 1/4/2010 |
| Lupus | Logan | chase | yes | 6/6/2014 |
| Lupus | Logan | displace | no | 6/17/2014 |

^a^ These males were only seen briefly, so identity is uncertain
