# Supplementary material for: Flanged males have higher reproductive success in a completely wild orangutan population
Source: PLoS One. 2024 Feb 9;19(2):e0296688. doi: 10.1371/journal.pone.0296688 (PMC10857694; doi:10.1371/journal.pone.0296688)
Supplement: S6 Table — (DOCX) [file pone.0296688.s006.docx]

**S6 Table. Rates at which male-female associations encounter an additional or extra-pair male (EPM), expressed as the average number of hours of association per encounter with an EPM**

|  | Sexually Active Female | Non-sexually Active Female | All females (combined) |
| --- | --- | --- | --- |
| Flanged male | 95.85 | 15.28 | 54.08 |
| Unflanged male | 70.78 | 43.08 | 56.65 |
| All males (combined) | 79.14 | 30.38 |  |
