# Supplementary material for: Flanged males have higher reproductive success in a completely wild orangutan population
Source: PLoS One. 2024 Feb 9;19(2):e0296688. doi: 10.1371/journal.pone.0296688 (PMC10857694; doi:10.1371/journal.pone.0296688)
Supplement: S7 Table — (DOCX) [file pone.0296688.s007.docx]

**S7 Table.** **GLMM testing the probability that a male-female pair encountered an extra-pair male (EPM)**

|  | Estimate | SE | z value | 95% CI | P value |
| --- | --- | --- | --- | --- | --- |
| *(Intercept)* | -1.836 | 0.589 | -3.117 | *-3.114; -0.723* | *0.002* |
| Predictor Variables: |  |  |  |  |  |
| UNF & NSA^a^ | -0.948 | 0.690 | -1.374 | -2.352; 0.421 | 0.169 |
| *FL & SA*^a^ | -1.973 | 0.561 | -3.514 | *-3.066; -0.839* | *<0.001* |
| *UNF & SA*^a^ | -1.405 | 0.657 | -2.138 | *-2.673; -0.042* | *0.033* |
| Control Variables: |  |  |  |  |  |
| *Fruit Availability* | 0.483 | 0.119 | 4.078 | *0.251; 0.719* | *<0.0001* |

^a^ Reference category: FL & NSA

UNF = Unflanged male; FL = Flanged male; NSA = Non-sexually active female; SA = Sexually active female

SE = standard error

CI = confidence interval

Significant effects are shown in *italics*

Random effects standard deviation for female ID = 0.1144 and male ID = 1.3099
