# Supplementary material for: Flanged males have higher reproductive success in a completely wild orangutan population
Source: PLoS One. 2024 Feb 9;19(2):e0296688. doi: 10.1371/journal.pone.0296688 (PMC10857694; doi:10.1371/journal.pone.0296688)
Supplement: S1 File — (DOCX) [file pone.0296688.s008.docx]

**S1 File. Reproductive skew calculation and Nonacs B interpretation**

Nonacs B index tells if measured skew is different than random skew. It is measured from -1 to +1, with -1 indicating an equal distribution, 0 indicating a random distribution, and +1 indicating monopolization. The Skew calculator 2013 also produces 95% confidence intervals, a minimum B value (the minimum B value possible given the dataset, indicating equal skew in the dataset), and a maximum B value (the maximum B value possible given the dataset, indicating monopolization in your dataset). If the 95% CI crosses zero, the possibility of a random distribution cannot be rejected. If the ‘equalB’ value is included in the 95% CI, an equal distribution cannot be rejected. If the ‘monopolB’ value is included in the 95% CI, complete monopolization cannot be rejected [79].

Because reproductive skew is expected to vary across different lengths of time, we then calculated the most successful sire’s share for 5-year intervals across the five orangutan populations with published paternity data. We used all 5-year periods where paternity data was known for at least two offspring. For the Sepilok data, we included data from 2010-2014; for the GPNP data, we included data from 2008-2014; for the Ketambe data, we included data from 1983-1997; for the Tanjung Puting data, we included data from 1993-2009; for the Kinabatangan data, we included data from six 5-year intervals from 1985-1995 and three 5-year intervals from 1993-2000.
